# Summer Diseases of Infants

**Published:** 1905-09

**Authors:** 


### PROGRESS IN MEDICAL SCIENCE.

### Summer Diseases of Infants.

### SUGGESTIONS FOR REDUCING THE PREVALENCE OF SUMMER DIARRHEA IN INFANTS.

T. S. Southworth (*Medical Record,* July 29, 1905,) says that a large part of the responsibility for the great infant mortality which recurs each summer rests on the medical profession, who have failed in their duty in anticipating such trouble by suitable prophylactic measures. These should date from the very birth of the child, and one of the most important is to urge breast nursing in place of bottle feeding. Over 90 per cent. of the deaths from gastrointestinal disturbances occur in bottle-fed infants, and it is safe to assert that the surest protection against the death of an infant from summer diarrhea lies in normal breast feeding. If the secretion of milk is scanty, it should be used for part of the feeding at least, and every effort should be made to encourage the flow. Much has already been accomplished in the way of educating the masses regarding the value of pure milk, but there is still a great deal to be done in this direction. Even after uncontaminated milk has been secured, however, it must be properly modified and kept cold, carelessness in the latter respect being sufficient to defeat the best intentions of the physician. Errors in weaning, neglect of apparently mild attacks of diarrhea, and the common diagnosis of teething, which is used as an excuse for almost any evidence of bodily derangement, are factors that must be combated. The sucking nipple is another distributor of infection that must be abolished. The physician's day's work, even if he sees a child but once, is to seek out and correct errors in nutrition, to combat popular misapprehensions, to further the use of clean milk, to warn the mother that at the very beginning of loose movements in summer she should stop the use of cow's milk in any form, clear out the bowels with castor oil, give water or cereal gruels only, and send promptly for the physician, since delay is so often fatal. Only through such personal, painstaking instruction of the masses can the desired end be accomplished.

---

### THE TREATMENT OF INFANTILE DIARRHEA.

In some important regards the treatment of diarrhea in infants is still far from occupying an unquestionable position. In a discussion of this subject in the leading societies one man will advocate the general employment of castor oil—a full dose—as an initial procedure; another looks upon such a method as unwise; one man will advise opium and some of the tannin preparations, another at once proclaims that opium and tannin have almost no place in the drug treatment of the disease: again one teacher strongly advocates the use of prepared foods, while an opponent vehemently states there is no "prepared" food worthy of the name, that they are all miserable—and worse than miserable—failures from a food standpoint, and that nothing, absolutely nothing, but the mother's milk should ever be used, when it is present and can be had, and when that is impossible the next thing, and natural substitute to a large degree, is diluted cow's milk. And thus the matter stands!

It would seem, however, "to the man up a tree," as if castor oil or calomel was plainly indicated where there was any reason to suspect remnants of undigested and unfit food in the intestinal canal; that opium has very great value, but must needs be used circumspectly; and that mother's milk has never yet been improved upon, while at the same time it often disagrees with an infant and must be, at least temporarily, withdrawn and an agreeable substitute furnished.

In carrying out these conditions and purposes no small degree of simon pure judgment—horse sense—is required, and a doctor cannot, in these cases any more than in others, always be directed by iron-bound rules and regulations.—*Clinical Review.*

---

### THE ANTISEPTIC TREATMENT OF THE SUMMER DIARRHEAS OF INFANTS.

Of the various agents that have been suggested for the disinfection of the intestinal tract, acetozone is by far the most promising. It has been shown by Novy and Freer, of the University of Michigan, that acetozone, even in weak solutions, destroys bacillus pyocyaneus, bacillus coli, bacillus typhosus, bacillus diphtheriæ, vibrio choleræ, staphylococcus pyogenes aureus, and streptococcus pyogenes, in less than one minute. These writers say that "while the strong solution kills everything almost instantly, the weaker solution (1-3000) destroys the vegetating germs, as a rule, within one minute." At the same time solutions of 1 to 1000 strength are given internally without the least harmful effect. The good results accruing from the use of this remedy in the summer complaints of young children are early and unmistakable; the discoloration and putridity of the stools disappear; the diarrhea is checked; the temperature falls; pain and inflammation subside; the vomiting is controlled; and the condition of anguish and irritability is consequently greatly dispelled.

In dealing with this class of cases the following make up the round of treatment: (a) withdrawal of milk and the substitution of thin broths, albumen and cereal waters, or other liquid feedings; (b) immediate evacuation of the stomach and intestines by stomach-washing and intestinal flushing with acetozone solution (1-5000 or stronger); (c) the sustaining of the patient's vitality: (d) administration of an internal antiseptic—acetozone (1-3000 to 1-1000); (e) the observance of hygienic conditions. In giving the drug, the solution usually administered to adults (15 grains to the quart) should be diluted with one-half its quantity of water and flavored with lemon or orange juice. It should be given in teaspoonful doses at frequent intervals—every twenty or thirty minutes in the beginning, lengthening the intervals as the case progresses.

Colonic irrigation is a useful procedure in cholera infantum. Acetozone (1-5000) solution is unexcelled for this purpose. The same solution may be used for lavage, which is recommended by many leading authorities. In washing out the stomach the irrigating fluid invariably should be luke warm and is best introduced prior to the feedings. Its continuance must be based on the character of the washings.

Acetozone is marketed in ounce, half-ounce, and quarter-ounce vials, and in boxes containing six vials of 15 grains each. An ounce is sufficient to make eight gallons of aqueous solution.

---

### COLONIC FLUSHING IN DIARRHEA.

Diarrheaic conditions all present certain physical signs which are in common, such as the frequent stools, great exhaustion and weakened heart action dependent upon the virulence of the poison. Recognising this toxic manifestation and the constantly increasing danger of autoinfection, the indicated treatment is one which will assist the effort nature is making to render the tract aseptic, by ridding itself of the contents of the bowel which has been a fertile field for disease-producing germs, to correct the existing fermentation and restore surface circulation which has been seriously interfered with. For this purpose a high colon flush of glycothymoline in a 10 per cent. solution at 105° should be used at least twice daily. This solution exerts a marked inhibitory effect upon the growth of putrefactive and pathogenic bacteria, it overcomes glandular stasis or edema present, depleting the membrane of its products of inflammation and restoring normal glandular secretion.

---

### "SHORTEN THE TIME FROM THE COW TO THE BABY."

ARTHUR R. REYNOLDS (*American Medicine,* October 22, 1904,) lays stress on the fact that twelve-hour milk is worth very much more from a dietetic standpoint than twenty-four-hour milk. Milk may be unfit food for the young many hours before it becomes sour to the taste. Milk begins to deteriorate, as to its digestibility and wholesomeness, from the moment it is exposed to the air. It is one of the best mediums for the growth and multiplication of bacteria. In twenty-four hours after being drawn, unless checked by cold, there will be 400,000 microörganisms in each teaspoonful of milk. Souring is due not only to the growth of bacteria which have fed and multiplied on the nutritive constituents of the milk, thereby reducing its food value, but to their production of a special souring (lactic) acid and other poisons. Old milk not only starves the young, but it poisons them. All milk intended for the use of children should be bottled in the country, immediately after having been thoroughly cooled. The bottles should be packed in broken ice and shipped to consumers within twelve hours after bottling. The writer declares that no legislation will be enacted making the delivery of twenty-four-hour to thirty-six-hour old milk illegal until the public is educated to a knowledge of the evils of stale milk and its murderous effects upon the young. The writer concludes his paper with the quotation from Lord Derby: "Sanitary education is more important than sanitary legislation."—*Medical Record.*

---

### NOVEL METHOD OF SAVING BABIES—MAYOR OF ENGLISH CITY PAYS PREMIUMS TO CAREFUL MOTHERS.

THE new mayor of Huddersfield, England, has promised to give to the mother of every child born during the year of his office a promissory note for £1, payable one year after birth, if the child lives so long. His object, briefly stated, is to prevent the wastage of life by making it worth while for the parents to be careful about their children. After considering the French system of offering a premium to children who had lived a year, he hit upon his present plan, because "the proper time to secure the child's welfare is to have the mother's help from the very first.

"I could not at first see quite how I could make a premium effective in preventing the child's death, which was what I wished to do. Then there came to me the happy thought of giving to the mother, so soon as the child was born, a properly legal promissory note, payable twelve months after date; and on the note I thought I might get in some good advice."

The promissory note is prepared in due legal form, and is accompanied by "The Golden Rule for Babies," the whole document being printed in colors and the shape of a certificate. The following is a copy of the note and the instructions following it:

FOR THE BABY.

Longwood District of the County Borough of Huddersfield.
Name of the Baby...............Date of Birth..............
Name and Address of Parents..............................

THE GOLDEN RULE.

For the life and health of the baby.

"Feed with the mother's milk; the mother's milk is the natural food and the best."

Twelve months after date I promise to pay to the parents or guardians of the above named child the sum of £1 on production of proof that the said child has reached the age of twelve months.

(Signed)........................
Mayor of Huddersfield.

For every baby fed on its mother's milk who dies before the age of three months fifteen babies die who have been fed by other means.

RULES FOR THE WELFARE OF THE BABY.

When the mother cannot suckle the child it should be fed on new milk and water, mixed in certain proportions, according to age.

At first half milk and half water, with a teaspoonful of cream and a little sugar. Then, as the child grows older, less water to be added. When cream cannot be obtained a small piece of suet may be shredded into the milk.

WHAT TO DO.

Always feed the baby at regular intervals every three hours.
Always keep the baby very clean.

Always bathe (or sponge all over) the baby once a day in warm water.

Always let the baby sleep in a cradle or cót; the wicker basket makes a good cot (or even an empty packing case).

Always use fuller's earth to powder the baby, not starch or flour.

Always attend to the baby when it cries. The baby cries for one of three reasons:

1. The baby is hungry; or
2. The baby is uncomfortable or something hurts; or
3. The baby is ill.

WHAT NOT TO DO.

Never give the baby soothing syrups, fever powders or anything of that sort.

Never give the baby bread or sops or gravy or any other food except milk till it is more than seven months old.

Never give the baby skimmed milk or milk that is not perfectly fresh and good.

Never use a feeding bottle with a long tube. Nobody can keep the inside of the tube clean.

Never carry the baby "sitting up" until it is five months old.

Never neglect to send for a doctor if the baby is ill. Babies are soon overcome, and easily die.—*The World.*

